# Inequality in the Survival of Patients With Head and Neck Cancer in Scotland

**DOI:** 10.3389/fonc.2018.00673

**Published:** 2019-01-22

**Authors:** Kate Ingarfield, Alex Douglas McMahon, Catriona M. Douglas, Shirley-Anne Savage, Kenneth MacKenzie, David I. Conway

**Affiliations:** ^1^University of Glasgow Dental School, University of Glasgow, Glasgow, United Kingdom; ^2^Department of Otolaryngology – Head and Neck Surgery, Queen Elizabeth University Hospital, Glasgow, United Kingdom; ^3^Emergency Care and Medicine Directorate, Victoria Hospital, Kirkcaldy, United Kingdom; ^4^Department of Otolaryngology – Head and Neck Surgery, Glasgow Royal Infirmary, Glasgow, United Kingdom

**Keywords:** head and neck cancer, socioeconomic status, survival, epidemiology, cohort, scotland, long term survival, deprivation

## Abstract

**Background:** Socioeconomic inequalities impact on the survival of head and neck cancer (HNC) patients, but there is limited understanding of the explanations of the inequality, particularly in long-term survival.

**Methods:** Patients were recruited from the Scottish Audit of Head and Neck Cancer between 1999 and 2001 and were linked to mortality data as at 30th September 2013. Socioeconomic status was determined using the area-based Carstairs 2001 index. Overall and disease-specific survival were calculated using the Kaplan-Meier method with 95% confidence intervals (CI's) at 1-, 5-, and 12-years. Net survival at 1-, 5-, and 12-years was also computed with 95% CIs. Cox proportional hazard models with 95% CIs were used to determine the explanations for the inequality in survival by all-cause mortality and disease-specific mortality with 95% CIs.

**Results:** Most patients were from the most deprived group, and were more likely to smoke, drink, have cancer of a higher stage and have a lower WHO Performance Status. A clear gradient across Carstairs fifths for unadjusted overall and disease-specific survival was observed at 1-, 5-, and 12-years for patients with HNC. Following the adjustment for multiple patient, tumor and treatment factors, the inequality in survival for patients with HNC had attenuated and was no longer statistically significant at 1-, 5-, and 12-years.

**Conclusion:** A clear gradient across Carstairs fifths for unadjusted overall, disease-specific and net survival was observed at 1-, 5-, and 12-years for HNC patients in Scotland from 1999 to 2001. This study concludes that explanations for the inequality in the survival of patients with HNC are not straightforward, and that many factors including various patient, tumor and treatment factors play a part in the inequality in the survival of patients with HNC.

## Introduction

Cancer survival often favors those who are from socioeconomically advantaged areas or have a more affluent backgrounds (1), and explanations for these socioeconomic inequalities are complex and difficult to explain. In the 1997 landmark IARC publication *Social Inequalities in Cancer*, Auvinen (2) assessed the socioeconomic factors that are associated with cancer survival, and identified research gaps in understanding the determinants of survival beyond cancer stage. Auvinen concluded that there was “urgent need” to understand the drivers of the inequality in cancer survival, and more than 20 years later, the evidence-base is not much further forward. Woods et al. (3) carried out a comprehensive review to determine the origins of socioeconomic inequalities in all-cancer survival and concluded that stage at diagnosis, access to health services, and comorbidity may explain some of the association.

Previous analyses of the Scottish Audit of Head and Neck Cancer (SAHNC) cohort have presented 5-year overall survival and 5-year disease-specific survival (4–7). In 2010, Robertson et al. (7) reported the impact of socioeconomic status (SES) on survival at 5-years and outlined that socioeconomic status was no longer an independent predictor of survival following the adjustment of multiple covariates. To add to this previous research, the aims of this study are to assess socioeconomic inequality in the survival of head and neck cancer patients assessing short-, mid-, and long-term survival, and to provide an understanding of the explanation of the inequality in the survival of these patients via different measurements of survival including overall, disease-specific and net survival estimates.

## Materials and Methods

### Patients

The SAHNC cohort recruited patients between 1st September 1999 and 31st August 2001—the methods have previously been described (4–7). Data were recorded on new HNC patients diagnosed in Scotland during this 2 year period. Quality assurance processes were carried out including cross-checking the data with medical and pathology results.

### Data Linkage and Approvals

The SAHNC cohort was linked to the National Records of Scotland (NRS) mortality data as at 30th September 2013 by ISD Scotland. Records were linked using an established probability matching technique based on the Howard Newcombe principle (8) which matches individual patients to their national Community Health Index (CHI) number—the unique healthcare identifier that is used in the National Health Service (NHS) in Scotland. Data were linked to mortality forms which outlined the primary and secondary causes of death using ICD10 codes. Information governance and data linkage approvals were obtained from the NHS Privacy Advisory Committee (now known as the Public Benefits and Privacy Panel).

### Measurement of Socioeconomic Status

SES was determined using the area-based Carstairs 2001 index (9, 10) which is defined from 2001 Census data consisting of four variables including proportion of unemployed males, those in social classes IV and V, those who do not own a car, and a measurement for overcrowding defined as a density of more than one person per room per private household. The Carstairs index is split at a population level by creating equal fifths using the quintile cut offs—group 1 represents the most affluent patients and group 5 represents the most deprived patients.

### Variables Used for Adjustment

Patient factors (age at diagnosis, sex, smoking behavior, alcohol consumption and patient performance status), tumor factors (stage and anatomical site) and treatment factors (treatment modality and geographic location of treatment) were collected at baseline and no further data was collected afterwards. Smoking behavior and alcohol consumption were determined from questionnaires at the time of diagnosis which allowed the following “tick-box” options for patient selection: “current smoker,” “previous smoker” and “never smoked,” and “current problem drinker,” “previous problem drinker” or “occasionally/never drinks”—no further data were collected on the patients' habits following diagnosis. Patient performance status was classified at diagnosis using the World Health Organization (WHO) Performance Status (11), which groups patients into one of five categories based on their level of physical abilities (“normal activity,” “strenuous activity restricted,” “up and about for more than 50% of waking hours,” “confined to a bed or chair for more than 50% of waking hours,” and “confined to a bed or chair for 100% of waking hours”). Tumor stage was determined using the Tumor, Node and Metastases (TNM) Classification of Malignant Tumors (12), and the cohort was grouped into stage I, II, III, or IV. Anatomical site was classified using the International Classification of Disease version 10 (13), grouped into seven categories—lip (C00.9), larynx (C32), nasal cavity (C11.9, C30.0, C31), oral cavity (C02–C04, C05.0, C06, C14), oropharynx (C01, C05.1–, C09, C10), hypopharynx (C12, C13), and other or salivary gland (C07, C08, C30.1, C41, C44, C76, C77). Treatment modality was grouped into five categories: (i) surgery only; (ii) radiotherapy only; (iii) surgery combined with radiotherapy; (iv) chemotherapy only, chemotherapy combined with surgery, chemotherapy combined with radiotherapy, and chemotherapy combined with both surgery and radiotherapy; and (v) no treatment. Location of treatment was based on the service delivered in the Scottish Cancer Networks located in three geographic region—West of Scotland Network (WoSCAN) (which comprises health board areas of Ayrshire and Arran, Forth Valley, Greater Glasgow, Clyde and Lanarkshire); South East Scotland Cancer Network (SCAN) (Borders, Dumfries and Galloway, Fife, Lothian); and North of Scotland Cancer Network (NOSCAN) (Grampian, Highland, Orkney, Shetland, Tayside, Western Isles).

### Statistical Methods

Overall and disease-specific survival were calculated using the Kaplan-Meier method with 95% confidence intervals (CI's). Overall survival considered all causes of death, whereas disease-specific survival only considered deaths where the patients' primary cause of death on their death certificate was a HNC ICD10 code. Cox proportional hazard models with 95% CIs were used to determine the explanations for the inequality in survival for all-cause mortality and disease-specific mortality with 95% CIs. Overall survival, disease-specific survival, and Cox proportional hazards models for all-cause mortality and disease-specific mortality were calculated using SAS Software, version 9.4 (SAS Institute Inc., USA). Net survival with 95% CIs was calculated by the Pohar-Perme method (14, 15) using the *stns* command in Stata 14 (16, 17), and using life-tables provided by the Cancer Survival Group at the London School of Tropical Hygiene and Medicine (18) These life-tables were standardized by age, sex, and Carstairs 2001 quintile. The Slope Index of Inequality (SII) was calculated based on the regression of the mid-point of survival for mortality for each SES group in each model (19).

## Results

### Cohort Recruitment

The SAHNC cohort recruited 77% (*n* = 1,910) of HNC cases that were diagnosed and recorded in the Scottish Cancer Registry over the study period from 1st September 1999 to 31st August 2001. Of the 1,910 patients in the baseline cohort, 1,895 were linked to 12-year mortality records−15 patients were excluded as they were unable to be matched to CHI numbers for data linkage follow-up. A further 15 patients were excluded as they could not be matched to Carstairs 2001 fifths, and 60 patients over the age of 85 were also excluded, which was a requirement for the successful computation of net survival for 12-year follow-up. A remaining 1,820 patients were included in the analyses.

### Patient Demographics by SES Carstairs 2001 Fifths

Table 1 outlines the demographic characteristics broken down by Carstairs 2001 fifths of the 1,820 patients. Most patients were from the most deprived regions of Scotland (29.0%), whereas only 13.2% of patients were from the most affluent areas of Scotland. There were no differences in the distribution of patients in each age category or between males and females across the Carstairs 2001 fifths. As deprivation increased from group 1 (most affluent) to group 5 (most deprived), the proportion of current smokers in each group also increased, and similarly, the proportion of patients with current alcohol problems increased. The number of patients experiencing normal WHO activity decreased across the SES groups, and the most deprived patients had the greatest proportion of Stage IV cancers (40.0%) compared to other groups. There was a slight difference in the treatment modalities used between groups, and the most deprived group had the smallest proportion of patients treated with curative intent. There were no clear trends by Carstairs 2001 fifths for anatomical site, Scottish Cancer Networks, or type of primary cause of death.

**Table 1:** Patient demographic, behavioral, tumor, and treatment characteristics by Carstairs quintiles.

**Variable**	**Total (Col. %)**	**Frequencies of Carstairs 2001 quintiles (Col. %)**					**Chi-square *p*-value**
		**1—Most affluent**	**2**	**3**	**4**	**5—Most deprived**	
Whole cohort (Row %)	1,820 (100.0%)	241 (13.2%)	317 (17.4%)	325 (17.9%)	409 (22.5%)	528 (29.0%)	–
**Age at diagnosis**							0.470
Less than 45	99 (5.4%)	16 (6.6%)	23 (7.3%)	16 (4.9%)	21 (5.1%)	23 (4.4%)	
45 to 54	288 (15.8%)	35 (14.5%)	44 (13.9%)	45 (13.9%)	68 (16.6%)	96 (18.2%)	
55 to 64	592 (32.5%)	70 (29.1%)	105 (33.1%)	108 (33.2%)	140 (34.2%)	169 (32.0%)	
65 to 74	551 (30.3%)	72 (29.9%)	90 (28.4%)	111 (34.2%)	108 (26.4%)	170 (32.2%)	
75 and over	290 (15.9%)	48 (19.9%)	55 (17.4%)	45 (13.9%)	72 (17.6%)	70 (13.3%)	
**Sex**							0.440
Male	1,300 (71.4%)	161 (66.8%)	227 (71.6%)	236 (72.6%)	289 (70.7%)	387 (73.3%)	
Female	520 (28.6%)	80 (33.2%)	90 (28.4%)	89 (27.4%)	120 (29.3%)	141 (26.7%)	
**Smoking status**							<0.001
Current smoker	1,134 (62.3%)	118 (49.0%)	173 (54.6%)	191 (58.8%)	256 (62.6%)	396 (75.0%)	
Previous smoker	405 (22.3%)	60 (24.9%)	86 (27.1%)	68 (20.9%)	100 (24.5%)	91 (17.2%)	
Never smoked	221 (12.1%)	56 (23.2%)	45 (14.2%)	50 (15.4%)	41 (10.0%)	29 (5.5%)	
Not recorded	60 (3.3%)	7 (2.9%)	13 (4.1%)	16 (4.9%)	12 (2.9%)	12 (2.3%)	
**Alcohol consumption**							<0.001
Current (problem) drinker	496 (27.3%)	51 (21.2%)	77 (24.3%)	80 (24.6%)	108 (26.4%)	180 (34.1%)	
Previous (problem) drinker	212 (11.7%)	25 (10.4%)	29 (9.2%)	49 (15.1%)	47 (11.5%)	62 (11.7%)	
Occasional/never drank	891 (49.0%)	138 (57.3%)	164 (51.7%)	150 (46.2%)	198 (48.4%)	241 (45.6%)	
Not recorded	221 (12.1%)	27 (11.2%)	47 (14.8%)	46 (14.2%)	56 (13.7%)	45 (8.5%)	
**WHO performance status**							0.003
Normal activity	825 (45.3%)	137 (56.9%)	169 (53.3%)	137 (42.3%)	177 (43.3%)	205 (38.8%)	
Strenuous activity restricted	465 (25.6%)	54 (22.4%)	66 (20.8%)	94 (28.9%)	102 (24.9%)	149 (28.2%)	
Up and about >50%	137 (7.5%)	18 (7.5%)	23 (7.3%)	17 (5.2%)	33 (8.1%)	46 (8.7%)	
Confined to bed/chair >50%	97 (5.3%)	8 (3.3%)	18 (5.7%)	22 (6.8%)	26 (6.4%)	23 (4.4%)	
Not recorded	296 (16.3%)	24 (10.0%)	41 (12.9%)	55 (16.9%)	71 (17.4%)	105 (19.9%)	
**Anatomical site**							0.470
Lip	85 (4.7%)	11 (4.6%)	17 (5.4%)	18 (5.5%)	23 (5.6%)	16 (3.0%)	
Larynx	584 (32.1%)	71 (29.5%)	102 (32.2%)	103 (31.7%)	143 (35.0%)	165 (31.3%)	
Nasal cavity	85 (4.7%)	12 (5.0%)	14 (4.4%)	22 (6.8%)	15 (3.7%)	22 (4.2%)	
Oral cavity	506 (27.8%)	76 (31.5%)	93 (29.3%)	78 (24.0%)	97 (23.7%)	162 (30.7%)	
Oropharynx	323 (17.8%)	40 (16.6%)	53 (16.7%)	63 (19.4%)	69 (16.9%)	98 (18.6%)	
Hypopharynx	119 (6.5%)	12 (5.0%)	19 (6.0%)	20 (6.2%)	35 (8.6%)	33 (6.3%)	
Other/salivary gland	118 (6.5%)	19 (7.9%)	19 (6.0%)	21 (6.5%)	27 (6.6%)	32 (6.1%)	
**Stage**							0.023
I	383 (21.0%)	58 (24.1%)	85 (26.8%)	75 (23.1%)	73 (17.9%)	92 (17.4%)	
II	369 (20.3%)	48 (19.9%)	62 (19.6%)	65 (20.0%)	88 (21.5%)	106 (20.1%)	
III	273 (15.0%)	37 (15.4%)	42 (13.3%)	40 (12.3%)	80 (19.6%)	74 (14.0%)	
IV	662 (36.4%)	79 (32.8%)	102 (32.2%)	125 (38.5%)	145 (35.5%)	211 (40.0%)	
Unknown	133 (7.3%)	19 (7.9%)	26 (8.2%)	20 (6.2%)	23 (5.6%)	45 (8.5%)	
**Treatment modality**							0.064
Surgery only	477 (26.2%)	72 (29.9%)	83 (26.2%)	86 (26.5%)	106 (25.9%)	130 (24.6%)	
Radiotherapy only	507 (27.9%)	74 (30.7%)	99 (31.2%)	98 (30.2%)	117 (28.6%)	119 (22.5%)	
Surgery + radiotherapy	458 (25.2%)	59 (24.5%)	82 (25.9%)	73 (22.5%)	101 (24.7%)	143 (27.1%)	
Chemo ± radio ± surgery	249 (13.7%)	23 (9.5%)	34 (10.7%)	48 (14.8%)	56 (13.7%)	88 (16.7%)	
No treatment	129 (7.1%)	13 (5.4%)	19 (6.0%)	20 (6.2%)	29 (7.1%)	48 (9.1%)	
**Network**							<0.001
WoSCAN (West Scotland)	1,001 (55.0%)	85 (35.3%)	110 (34.7%)	149 (45.9%)	244 (59.7%)	413 (78.2%)	
SCAN (East Scotland)	440 (24.2%)	83 (34.4%)	85 (26.8%)	108 (33.2%)	108 (26.4%)	56 (10.6%)	
NOSCAN (North Scotland)	379 (20.8%)	73 (30.3%)	122 (38.5%)	68 (20.9%)	68 (20.9%)	59 (11.2%)	
**Treatment intent**							0.053
Curative	1,355 (74.5%)	196 (81.3%)	250 (78.9%)	239 (73.5%)	307 (75.1%)	363 (68.8%)	
Palliative	307 (16.9%)	29 (12.0%)	42 (13.3%)	61 (18.7%)	69 (16.9%)	106 (20.1%)	
unknown	158 (8.7%)	16 (6.6%)	25 (7.9%)	25 (7.7%)	33 (8.1%)	59 (11.2%)	
**Primary cause of death**							0.063
Cancer—Head and neck	677 (37.2%)	78 (32.4%)	99 (31.2%)	127 (39.1%)	157 (38.4%)	216 (21.6%)	
Cancer—Other	308 (16.9%)	46 (19.1%)	54 (17.0%)	61 (18.8%)	73 (17.8%)	74 (14.0%)	
Other/unknown	399 (21.9%)	59 (24.5%)	73 (23.0%)	59 (18.5%)	81 (19.8%)	126 (23.9%)	
Alive	436 (24.0%)	58 (24.1%)	91 (28.7%)	77 (23.7%)	98 (24.0%)	112 (21.2%)	

### Overall, Disease-Specific, and Net Survival

One-, five-, and twelve-year overall, disease-specific and net survival by Carstairs 2001 fifths are displayed in Table 2. The Slope Index of Inequality (SII) for all three methods of survival and at each time point are also displayed in Table 2. One-year overall survival for the most deprived patients was 71.8% (67.7%, 75.4%), whereas the most affluent patients' was higher at 83.4% (78.1%, 87.5%). By 5-years, the inequality remained the same, and by 12-years the difference in overall survival had reduced, which can be demonstrated by a similar SII at 5-year [SII = 12.9 (−1.8, 27.5)] and a reduced SII at 12-years [SII = 7.4 (−2.7, 17.5)] compared to the SII at 1-year 12.7 (6.7, 18.8). One-year disease-specific survival for the most deprived patients was 79.1% (75.2%, 82.4%) whereas the most affluent patients' was higher at 88.8% (83.9%, 92.2%). By 5- and 12-years, the gap between the most affluent and most deprived patients for disease-specific survival had widened, and the SII had increased from 9.5 (1.4, 17.7) at 1-year to 12.5 (−1.8, 26.9) and 16.5 (1.5, 31.5) at 5- and 12-years, respectively. Net survival at 1 year for the most deprived patients was 73.7% (69.7%, 77.6%), compared to the most affluent patients at 86.1% (81.3%, 91.0%). The inequality between the net survival results for the most deprived patients and the most affluent patients was strong at 1-year with SII of 13.6 (7.1, 20.1), however this had widened by 5-years with SII of 16.1 (−1.0, 33.3), and by 12-years a weak inequality remained with the SII at 6.6 (−17.2, 30.3).

**Table 2:** Overall and disease-specific survival at One-, five-, and twelve-years by Carstairs 2001 quintiles for all patients.

	**1-year**	***p*-value**	**5-years**	***p*-value**	**12-years**	***p*-value**
**OVERALL SURVIVAL**						
Whole cohort	76.0 (74.0, 77.9)	–	46.1 (43.8, 48.4)	–	26.3 (24.3, 28.3)	–
Carstairs quintile		0.007		0.002		0.010
1 (Most affluent)	83.4 (78.1, 87.5)		49.8 (43.3, 55.9)		27.0 (21.5, 32.7)	
2	78.6 (73.6, 82.7)		52.1 (46.4, 57.4)		30.6 (25.6, 35.7)	
3	76.3 (71.3, 80.6)		44.6 (39.2, 49.9)		26.2 (21.5, 31.0)	
4	75.1 (70.6, 79.0)		47.7 (42.8, 52.4)		26.9 (22.7, 31.3)	
5 (Most deprived)	71.8 (67.7, 75.4)		40.5 (36.3, 44.7)		22.9 (19.4, 26.6)	
Slope Index of Inequality (95% CIs)	12.7 (6.7, 18.8)		12.9 (−1.8, 27.5)		7.4 (−2.7, 17.5)
**DISEASE-SPECIFIC SURVIVAL**						
Whole cohort	82.3 (80.4, 84.0)	–	64.1 (61.7, 66.4)	–	56.9 (54.3, 59.4)	–
Carstairs quintile		0.031		0.009		0.003
1 (Most affluent)	88.8 (83.9, 92.2)		69.6 (62.9, 75.3)		61.8 (54.4, 68.4)	
2	83.2 (78.5, 86.9)		69.8 (64.2, 74.8)		65.6 (59.6, 70.9)	
3	82.2 (77.5, 86.1)		61.0 (55.1, 66.4)		55.5 (49.2, 61.3)	
4	81.8 (77.5, 85.3)		64.4 (59.2, 69.1)		55.5 (49.9, 60.8)	
5 (Most deprived)	79.1 (75.2, 82.4)		59.6 (54.9, 63.9)		51.1 (46.0, 55.9)	
Slope Index of Inequality (95% CIs)	9.5 (1.4, 17.7)		12.5 (−1.8, 26.9)		16.5 (1.5, 31.5)	
**NET SURVIVAL**						
Whole cohort	78.3 (76.2, 80.3)	–	53.9 (51.1, 56.6)	–	41.4 (37.7, 45.1)	–
Carstairs quintile		N/A^*^		N/A^*^		N/A^*^
1 (Most affluent)	86.1 (81.3, 91.0)		58.1 (50.4, 65.8)		40.4 (30.7, 50.0)	
2	80.9 (76.2, 85.5)		61.0 (54.4, 67.6)		43.8 (35.0, 52.6)	
3	78.6 (73.8, 83.3)		52.9 (46.4, 59.3)		40.7 (31.5, 49.9)	
4	77.2 (72.8, 81.5)		55.8 (50.1, 61.6)		46.6 (38.4, 54.7)	
5 (Most deprived)	73.7 (69.7, 77.6)		46.6 (41.7, 51.5)		35.7 (29.6, 41.8)	
Slope Index of Inequality (95% CIs)	13.6 (7.1, 20.1)		16.1 (−1.0, 33.3)		6.6 (−17.2, 30.3)

* *Trend test does not exist for Net survival*.

### Cox Models for All-Cause Mortality

Minimally adjusted and fully adjusted Cox Proportional Hazards models for all-cause mortality are displayed in Table 3. Clear trends can be observed following minimal adjustment by age and sex in the models for all-cause mortality at all three time points, and there is statistical evidence to confirm an inequality in all-cause mortality at 1- (*p* < 0.001), 5- (*p* < 0.001), and 12-years (*p* < 0.001). At 1 year, the most deprived patients were 46% more at risk when the model was adjusted by age, sex and patient factors [HR 1.46, (1.02, 2.09)], and there was evidence of a difference between the most affluent patients and those who were in the most deprived group (*p* = 0.037). Following the adjustment for age, sex, tumor and treatment factors, there was no longer any evidence of a difference between the most affluent patients and the patients in other Carstairs 2001 fifth (*p* = 0.113), and this result was also clear when the model was fully adjusted by age, sex, patient, tumor and treatment factors (*p* = 0.351). This was also demonstrated by the SII's which had reduced from 1.1 (0.7, 1.5) in the minimally adjusted model by age and sex, to 0.2 (−0.4, 0.7) in the fully adjusted model. By 5- and 12-years, the gaps between the risk of all-cause mortality for the most affluent and the most deprived patients had narrowed in all models, which can be demonstrated by a reduction in all the models' SIIs between 1-, 5-, and 12-year follow-up—for example, in the model that was minimally adjusted by age and sex, the SII had reduced from 1.1 (0.7, 1.5) at 1-year, to 0.6 (0.1, 1.0) at 5-years, and 0.4 (0.1, 0.7) at 12-years, whereas in the fully adjusted model the SII had reduced from 0.2 (−0.4, 0.7) at 1-year, to 0.03 (−0.6, 0.6) at 5-years, and −0.1 (−0.5, 0.4) at 12-years. There was no longer any evidence of an inequality by all-cause mortality by 5- or 12-years following the adjustment for age, sex and patient, tumor or treatment factors.

**Table 3:** One-, five-, and twelve-year all-cause mortality (ACM) and disease-specific mortality (DSM) hazard ratios by Carstairs 2001 quintile for all patients with slope index of inequality (SII) for each measurement and time point.

**Variable**	**Adjusted by age and sex**		Adjusted by patient factors^*^		**Adjusted by tumor and treatment factors∧**		**Adjusted by patient, tumor and treatment factors+**	
	**HR (95% CIs)**	***p*-value**	**HR (95% CIs)**	***p*-value**	**HR (95% CIs)**	***p*-value**	**HR (95% CIs)**	***p*-value**
**1-year ACM**		<0.001		0.037		0.113		0.351
1 (Most affluent)	1.00 (Ref.)		1.00 (Ref.)		1.00 (Ref.)		1.00 (Ref.)	
2	1.35 (0.92, 2.00)		1.20 (0.81, 1.78)		1.09 (0.74, 1.62)		0.99 (0.67, 1.47)	
3	1.53 (1.05, 2.25)		1.31 (0.89, 1.92)		1.42 (0.96, 2.09)		1.28 (0.86, 1.89)	
4	1.62 (1.12, 2.33)		1.30 (0.90, 1.89)		1.29 (0.89, 1.88)		1.16 (0.79, 1.69)	
5 (Most deprived)	1.96 (1.38, 2.77)		1.46 (1.02, 2.09)		1.32 (0.92, 1.91)		1.16 (0.80, 1.68)	
SII (95% CIs)	1.1 (0.7, 1.5)		0.5 (0.2, 0.8)		0.3 (−0.3, 1.0)		0.2 (−0.4, 0.7)	
**5-year ACM**		<0.001		0.157		0.065		0.715
1 (Most affluent)	1.00 (Ref.)		1.00 (Ref.)		1.00 (Ref.)		1.00 (Ref.)	
2	0.99 (0.78, 1.26)		0.89 (0.70, 1.13)		0.91 (0.72, 1.16)		0.80 (0.63, 1.02)	
3	1.22 (0.97, 1.54)		1.07 (0.85, 1.35)		1.29 (1.02, 1.63)		1.11 (0.88, 1.41)	
4	1.14 (0.91, 1.43)		0.95 (0.76, 1.19)		1.07 (0.85, 1.34)		0.90 (0.72, 1.14)	
5 (Most deprived)	1.43 (1.15, 1.76)		1.11 (0.89, 1.38)		1.17 (0.94, 1.46)		0.97 (0.78, 1.22)	
SII (95% CIs)	0.6 (0.1, 1.0)		0.2 (−0.2, 0.6)		0.2 (−0.5, 0.9)		0.03 (−0.6, 0.6)	
**12-year ACM**		<0.001		0.624		0.197		0.465
1 (Most affluent)	1.00 (Ref.)		1.00 (Ref.)		1.00 (Ref.)		1.00 (Ref.)	
2	0.94 (0.77, 1.15)		0.86 (0.70, 1.04)		0.87 (0.71, 1.06)		0.79 (0.64, 0.96)	
3	1.09 (0.89, 1.32)		0.95 (0.78, 1.16)		1.14 (0.94, 1.39)		0.99 (0.81, 1.21)	
4	1.08 (0.89, 1.30)		0.89 (0.74, 1.08)		1.02 (0.84, 1.23)		0.86 (0.71, 1.05)	
5 (Most deprived)	1.27 (1.06, 1.52)		0.99 (0.82, 1.19)		1.06 (0.88, 1.27)		0.87 (0.72, 1.05)	
SII (95% CIs)	0.4 (0.1, 0.7)		0.1 (−0.3, 0.4)		0.1 (−0.4, 0.6)		−0.1 (−0.5, 0.4)	
**1-year DSM**		0.001		0.162		0.129		0.431
1 (Most affluent)	1.00 (Ref.)		1.00 (Ref.)		1.00 (Ref.)		1.00 (Ref.)	
2	1.59 (0.99, 2.54)		1.41 (0.88, 2.27)		1.23 (0.76, 1.97)		1.11 (0.69, 1.80)	
3	1.69 (1.06, 2.69)		1.42 (0.88, 2.27)		1.57 (0.98, 2.52)		1.40 (0.87, 2.26)	
4	1.72 (1.10, 2.69)		1.37 (0.87, 2.16)		1.42 (0.90, 2.25)		1.24 (0.78, 1.98)	
5 (Most deprived)	2.09 (1.36, 3.22)		1.51 (0.97, 2.34)		1.45 (0.92, 2.29)		1.23 (0.78, 1.96)	
SII (95% CIs)	1.1 (0.2, 1.9)		0.7 (−0.1, 3.5)		0.4 (−0.4, 1.3)		0.2 (−0.5, 0.9)	
**5-year DSM**		<0.001		0.117		0.046		0.343
1 (Most affluent)	1.00 (Ref.)		1.00 (Ref.)		1.00 (Ref.)		1.00 (Ref.)	
2	1.06 (0.77, 1.46)		0.96 (0.70, 1.33)		0.94 (0.68, 1.29)		0.82 (0.59, 1.14)	
3	1.41 (1.04, 1.92)		1.24 (0.91, 1.68)		1.49 (1.10, 2.03)		1.29 (0.95, 1.77)	
4	1.28 (0.95, 1.72)		1.07 (0.79, 1.45)		1.21 (0.89, 1.63)		1.03 (0.75, 1.40)	
5 (Most deprived)	1.55 (1.17, 2.06)		1.21 (0.91, 1.62)		1.27 (0.95, 1.71)		1.07 (0.79, 1.45)	
SII (95% CIs)	0.7 (0.1, 1.2)		0.3 (−0.3, 0.8)		0.3 (−0.7, 1.4)		0.1 (−0.7, 1.0)	
**12-year DSM**		<0.001		0.066		0.036		0.359
1 (Most affluent)	1.00 (Ref.)		1.00 (Ref.)		1.00 (Ref.)		1.00 (Ref.)	
2	0.98 (0.73, 1.33)		0.89 (0.66, 1.20)		0.88 (0.65, 1.19)		0.78 (0.58, 1.06)	
3	1.32 (0.99, 1.75)		1.16 (0.87, 1.54)		1.38 (1.04, 1.85)		1.20 (0.90, 1.61)	
4	1.27 (0.97, 1.68)		1.06 (0.80, 1.40)		1.19 (0.90, 1.58)		1.02 (0.77, 1.35)	
5 (Most deprived)	1.51 (1.16, 1.96)		1.18 (0.90, 1.55)		1.22 (0.92, 1.60)		1.02 (0.77, 1.35)	
SII (95% CIs)	0.7 (0.2, 1.2)		0.3 (−0.2, 0.7)		0.3 (−0.6, 1.2)		0.1 (−0.7, 0.9)	

* *Adjusted by age, sex, and patient factors including smoking status, alcohol consumption and WHO Performance Status*.

*^∧^Adjusted by age, sex, tumor and treatment factors including stage, anatomical site, treatment modality and network of treatment ^+^Adjusted by all factors including smoking status, alcohol consumption, WHO Performance status, stage, anatomical site, treatment modality and network of treatment*.

### Cox Models for Disease-Specific Mortality

Minimally adjusted and fully adjusted Cox Proportional Hazards models for disease-specific mortality are displayed in Table 3. Similar to the models for all-cause mortality, there were clear trends following minimal adjustment by age and sex in the models for disease-specific mortality at all three time points, and there is statistical evidence to confirm an inequality in disease-specific mortality at 1- (*p* = 0.001), 5- (*p* < 0.001), and 12-years (*p* < 0.001). Following full adjustment for all factors including age, sex, patient, tumor and treatment factors, there was no evidence to support an inequality in excess risk of disease-specific mortality after 1-year (*p* = 0.431), which can also be demonstrated by the SII which had reduced from 1.1 (0.2, 1.9) in the model minimally adjusted by age and sex, to 0.2 (−0.5, 0.9) in the fully adjusted model. By 5- and 12-years, the gaps between the risk of disease-specific mortality for the most affluent and the most deprived patients had narrowed in all models, which can be demonstrated by a reduction in all the models' SIIs between 1-, 5-, and 12-year follow-up—for example, in the model that was minimally adjusted by age and sex, the SII had reduced from 1.1 (0.2, 1.9) at 1-year, to 0.7 (0.1, 1.2) at 5-years, to 0.7 (0.2, 1.2) at 12-years, whereas in the fully adjusted model the SII had reduced from 0.2 (−0.5, 0.9) at 1-year, to 0.1 (−0.7, 1.0) at 5-years, and −0.1 (−0.5, 0.9) at 12-years. There was statistical evidence of a difference in the risk of disease-specific mortality at 5- and 12-years in the models adjusted by age, sex, tumor and treatment factors which is determined by the patients in group 3 (intermediate affluency) having 49% excess risk of disease-specific mortality [HR = 1.49, (1.10, 2.03)] and 38% excess risk of disease-specific mortality [HR = 1.38, (1.04, 1.85)] at 5- and 12-years, respectively, compared to those who were in the most affluent group. However, there was no evidence of an inequality across the groups from the SIIs at 5- or 12-years [SII = 0.3, (−0.7, 1.4] and 0.3, (−0.6, 1.2, respectively).

## Discussion

This study demonstrates a clear gradient across Carstairs quintiles for minimally adjusted overall, disease-specific and net survival at 1-, 5-, and 12-years for patients with a diagnosis of HNC made between the years of 1999 and 2001 from Scotland. Following full adjustment at 1-, 5-, and 12-years, the inequality was no longer statistically significant suggesting that the inequality in the survival of patients with HNC can be explained by multiple patient, tumor and treatment factors. As an additional analysis, we also investigated the impact of individual co-variates on the inequality in survival, but the inequality remained strong at all three time points for all-cause mortality and disease-specific mortality (Supplementary Tables 1, 2), suggesting that the inequality in the survival of patients with HNC is not straightforward, and many factors play a combined effect in the role of the explanation of the inequality in HNC survival. The results for the net survival (unadjusted) analysis demonstrated a clear gradient across the Carstairs fifths at 1- and 5-years, but this gradient disappears by 12-years, suggesting that some of the inequality in long-term survival is partly attributable to background mortality, and since this cohort has such long follow-up, influence from background mortality is to be expected.

There are several limitations to this study. Firstly, SES was measured using the area based Carstairs 2001 Index (9, 10), which is derived from 2001 Census data involving the proportion of male unemployment, those in social classes IV and V, lack of car ownership, and overcrowding in a dwelling. Since this was a clinical cohort study, further data on SES indexes including education level and amount of income, was not collected as part of this study. Carstairs 2001 Index may not accurately represent rural and urban populations as it may be essential for people in these areas to own a car, however as other indices such as education level of income were not available for this analysis, Carstairs 2001 scores were the best measurement available for this analysis.

A further limitation of this study is the use of disease-specific survival which was classified from a patient having a primary cause of death of a form of HNC on their death certificates. Death certificates often contain several causes of death and so an exact cause of patients' death is not usually possible to determine, therefore we advise that these results are interpreted with caution due to the reliability of the reporting of cause of death from death certificates. Due to this, we have included net survival estimates alongside overall and disease-specific survival results to give an additional representation of HNC-specific deaths. Net survival determines the excess hazard of death from HNC, and therefore the impact of background mortality in HNC survival can be assessed. However, net survival cannot be computed in Cox Proportional Hazard analyses to run adjustment for additional confounders, and so all-cause and disease-specific mortality models together with net survival estimates provide a thorough insight into the burden of disease in HNC patients.

There has been an increase in the incidence of HPV-associated HNC over the last 20 years (20–22), which mostly involves cancers of the oropharynx. Around two-thirds of oropharynx cancers may be explained by HPV (23), and patients have substantially better prognoses than those with non-HPV-driven tumors, suggesting that one limitation of this study is the absence of HPV data (24, 25). These data were collected between the years of 1999 and 2001, which was before the discovery of the significant difference in survival between HPV-positive and HPV-negative HNC tumors (26), and thus HPV data was not collected as part of this study. Smoking and alcohol consumption are the main risk factors for non-oropharyngeal HNC tumors, and apart from tumors of the oropharynx, most HNCs are HPV-negative (27, 28). The marked improvement of in the survival of patients with HPV-positive oropharyngeal tumors was not observed in this study (data not shown), suggesting that these tumors are likely to be HPV-negative and therefore mostly explained by the high prevalence of smoking and alcohol consumption in this cohort (27, 28). HPV status, smoking behavior and alcohol consumption are three independent risk factors of survival (29, 30), and therefore we believe that the majority of cancers in this study are smoking and alcohol related and thus we believe that our findings are relevant despite missing HPV data.

Socioeconomic inequalities are present in HNC survival and are observed between and within countries. There are global inequalities in the incidence and mortality of HNC, and around two thirds of cases and three-quarters of deaths occur in low- or middle-income countries^1^. Paterson et al. (31) reported that the initial differences in survival (up to 18 months from diagnosis) may be explained by an advanced stage at diagnosis in the patients who were most deprived, and once this effect was eliminated, deprivation was no longer a predictor of patient prognosis for those who survive beyond 18 months. Ellis et al. (32) confirmed that there was a gap in relative survival by deprivation at both 1 and 5 years in favor of the patients from socioeconomically advantaged areas and concluded that the origins of the inequalities were unclear, although it was likely that comorbidities and healthcare access were contributing toward the differences.

This study adds to the understanding of the inequality in survival for head and neck cancer patients. The SAHNC cohort represented 77% of all HNC cases on the Scottish Cancer Registry over a 2-year period and is therefore a good representation of HNC cases in Scotland. In unadjusted models, a clear gradient across Carstairs quintiles for overall, disease-specific and net survival was observed at 1-, 5-, and 12-years for this cohort of HNC patients. Following adjustment for multiple patient, tumor and treatment factors the inequality was no longer present for all-cause and disease-specific mortality. This study concludes that explanations for the inequality in survival of patients with HNC are not straightforward. Many factors, including various patient, tumor and treatment factors, play a part in the inequality of survival in patients with HNC.

## Ethics Statement

Research ethics committee advice was sought using the online tool from the NHS health research authority and Medical Research council website and was not required.

## Author Contributions

KI statistical analysis and wrote paper; AM statistical analysis and supervision; DC supervision; CD supervision; KM supervision; S-AS supervision.

### Conflict of Interest Statement

The authors declare that the research was conducted in the absence of any commercial or financial relationships that could be construed as a potential conflict of interest.

## Footnotes

^1^http://publications.iarc.fr/Non-Series-Publications/World-Cancer-Reports/World-Cancer-Report-2014
