# Supplementary material for: Inequality in the Survival of Patients With Head and Neck Cancer in Scotland
Source: Front Oncol. 2019 Jan 22;8:673. doi: 10.3389/fonc.2018.00673 (PMC6349751; doi:10.3389/fonc.2018.00673)
Supplement: Supplementary file 1 [file Data_Sheet_1.docx]

Supplementary Table 1 – All-cause mortality (ACM) and disease-specific mortality (DSM) hazard ratios adjusted by patient factors for all patients

|  | **Adjusted by WHO*** | | **Adjusted by Smoking^** | | **Adjusted by Alcohol+** | | **Adjusted by Site#** | | **Adjusted by Stage$** | | **Adjusted by Treatment&** | | **Adjusted by Network=** | |
| --- | --- | --- | --- | --- | --- | --- | --- | --- | --- | --- | --- | --- | --- | --- |
| **Variable** | **HR (95% CIs)** | **p-value** | **HR (95% CIs)** | **p-value** | **HR (95% CIs)** | **p-value** | **HR (95% CIs)** | **p-value** | **HR (95% CIs)** | **p-value** | **HR (95% CIs)** | **p-value** | **HR (95% CIs)** | **p-value** |
| **One-year ACM** |  | 0.003 |  | 0.003 |  | <0.001 |  | <0.001 |  | 0.002 |  | 0.007 |  | <0.001 |
| 1 (Most affluent) | 1.00 (Ref.) |  | 1.00 (Ref.) |  | 1.00 (Ref.) |  | 1.00 (Ref.) |  | 1.00 (Ref.) |  | 1.00 (Ref.) |  | 1.00 (Ref.) |  |
| 2 | 1.22 (0.83, 1.81) |  | 1.31 (0.88, 1.93) |  | 1.36 (0.92, 2.01) |  | 1.38 (0.93, 2.03) |  | 1.32 (1.89, 1.95) |  | 1.21 (0.81, 1.78) |  | 1.36 (0.92, 2.01) |  |
| 3 | 1.38 (0.94, 2.03) |  | 1.47 (1.00, 2.15) |  | 1.51 (1.03, 2.22) |  | 1.54 (1.05, 2.25) |  | 1.51 (1.03, 2.21) |  | 1.53 (1.04, 2.25) |  | 1.53 (1.04, 2.25) |  |
| 4 | 1.37 (0.95, 1.98) |  | 1.49 (1.03, 2.15) |  | 1.57 (1.09, 2.27) |  | 1.63 (1.13, 2.35) |  | 1.52 (1.05, 2.20) |  | 1.48 (1.03, 2.14) |  | 1.61 (1.12, 2.33) |  |
| 5 (Most deprived) | 1.66 (1.16, 2.35) |  | 1.68 (1.18, 2.40) |  | 1.85 (1.30, 2.63) |  | 1.91 (1.35, 2.71) |  | 1.73 (1.22, 2.46) |  | 1.58 (1.11, 2.25) |  | 1.96 (1.37, 2.80) |  |
| *SII (95% CIs)* | *0.7 (0.3, 1.1)* |  | *0.7 (0.2, 1.2)* |  | *0.9 (0.4, 1.4)* |  | *1.0 (0.5, 1.5)* |  | *0.8 (0.3, 1.3)* |  | *0.6 (0.0, 1.3)* |  | *1.7 (0.6, 1.5)* |  |
| **Five-year ACM** |  | 0.008 |  | 0.012 |  | 0.001 |  | <0.001 |  | 0.004 |  | 0.005 |  | <0.001 |
| 1 (Most affluent) | 1.00 (Ref.) |  | 1.00 (Ref.) |  | 1.00 (Ref.) |  | 1.00 (Ref.) |  | 1.00 (Ref.) |  | 1.00 (Ref.) |  | 1.00 (Ref.) |  |
| 2 | 0.90 (0.71, 1.14) |  | 0.97 (0.76, 1.23) |  | 1.00 (0.79, 1.27) |  | 1.03 (0.81, 1.31) |  | 1.00 (0.79, 1.27) |  | 0.91 (0.72, 1.16) |  | 0.99 (0.78, 1.26) |  |
| 3 | 1.12 (0.89, 1.41) |  | 1.19 (0.94, 1.49) |  | 1.21 (0.96, 1.52) |  | 1.28 (1.02, 1.61) |  | 1.25 (0.99, 1.57) |  | 1.23 (0.97, 1.55) |  | 1.22 (0.96, 1.53) |  |
| 4 | 1.00 (0.80, 1.25) |  | 1.07 (0.85, 1.34) |  | 1.10 (0.88, 1.38) |  | 1.19 (0.95, 1.49) |  | 1.10 (0.88, 1.37) |  | 1.12 (0.89, 1.40) |  | 1.13 (0.90, 1.42) |  |
| 5 (Most deprived) | 1.24 (1.00, 1.53) |  | 1.26 (1.02, 1.56) |  | 1.35 (1.09, 1.67) |  | 1.41 (1.15, 1.75) |  | 1.32 (1.07, 1.63) |  | 1.24 (1.00, 1.53) |  | 1.40 (1.13, 1.74) |  |
| *SII (95% CIs)* | *0.3 (-0.2, 0.9)* |  | *0.3 (-0.1, 0.8)* |  | *0.4 (0.0, 0.9)* |  | *0.5 (0.1, 0.9)* |  | *0.4 (-0.1, 0.9)* |  | *0.3 (-0.2, 0.9)* |  | *0.5 (0.1, 1.0)* |  |
| **12-year ACM** |  | 0.029 |  | 0.102 |  | 0.009 |  | 0.001 |  | 0.012 |  | 0.010 |  | 0.006 |
| 1 (Most affluent) | 1.00 (Ref.) |  | 1.00 (Ref.) |  | 1.00 (Ref.) |  | 1.00 (Ref.) |  | 1.00 (Ref.) |  | 1.00 (Ref.) |  | 1.00 (Ref.) |  |
| 2 | 0.87 (0.71, 1.06) |  | 0.92 (0.75, 1.12) |  | 0.95 (0.78, 1.16) |  | 0.96 (0.79, 1.18) |  | 0.94 (0.77, 1.15) |  | 0.88 (0.72, 1.07) |  | 0.93 (0.77, 1.14) |  |
| 3 | 1.01 (0.83, 1.23) |  | 1.05 (0.87, 1.28) |  | 1.06 (0.88, 1.29) |  | 1.14 (0.94, 1.39) |  | 1.09 (0.90, 1.33) |  | 1.10 (0.90, 1.33) |  | 1.08 (0.89, 1.31) |  |
| 4 | 0.96 (0.79, 1.16) |  | 1.00 (0.83, 1.20) |  | 1.04 (0.86, 1.25) |  | 1.12 (0.93, 1.35) |  | 1.04 (0.86, 1.25) |  | 1.07 (0.89, 1.29) |  | 1.05 (1.05, 1.27) |  |
| 5 (Most deprived) | 1.13 (0.94, 1.35) |  | 1.10 (0.92, 1.32) |  | 1.20 (1.01, 1.44) |  | 1.25 (1.05, 1.50) |  | 1.19 (1.00, 1.42) |  | 1.14 (0.96, 1.37) |  | 1.22 (1.02, 1.47) |  |
| *SII (95% CIs)* | *0.2 (-0.2, 0.6)* |  | *0.2 (-0.1, 0.4)* |  | *0.3 (0.0, 0.6)* |  | *0.3 (0.1, 0.6)* |  | *0.3 (0.0, 0.6)* |  | *0.3 (-0.1, 0.6)* |  | *0.3 (0.0, 0.6)* |  |
| **One-year DSM** |  | 0.025 |  | 0.026 |  | 0.005 |  | 0.002 |  | 0.012 |  | 0.039 |  | <0.001 |
| 1 (Most affluent) | 1.00 (Ref.) |  | 1.00 (Ref.) |  | 1.00 (Ref.) |  | 1.00 (Ref.) |  | 1.00 (Ref.) |  | 1.00 (Ref.) |  | 1.00 (Ref.) |  |
| 2 | 1.42 (0.88, 2.28) |  | 1.54 (0.96, 2.47) |  | 1.60 (1.00, 2.56) |  | 1.61 (1.01, 2.59) |  | 1.55 (0.97, 2.49) |  | 1.42 (0.88, 2.27) |  | 1.58 (0.98, 2.53) |  |
| 3 | 1.50 (0.94, 2.39) |  | 1.62 (1.01, 2.59) |  | 1.67 (1.05, 2.67) |  | 1.69 (1.06, 2.70) |  | 1.68 (1.05, 2.69) |  | 1.73 (1.08, 2.77) |  | 1.71 (1.07, 2.72) |  |
| 4 | 1.43 (0.91, 2.25) |  | 1.59 (1.01, 2.49) |  | 1.67 (1.07, 2.62) |  | 1.74 (1.11, 2.72) |  | 1.63 (1.04, 2.56) |  | 1.60 (1.02, 2.51) |  | 1.77 (1.13, 2.79) |  |
| 5 (Most deprived) | 1.73 (1.12, 2.67) |  | 1.78 (1.15, 2.75) |  | 1.98 (1.28, 3.05) |  | 2.05 (1.33, 3.15) |  | 1.86 (1.21, 2.87) |  | 1.68 (1.08, 2.59) |  | 2.21 (1.42, 3.44) |  |
| *SII (95% CIs)* | *0.7 (0.0, 1.4)* |  | *0.7 (-0.2, 1.6)* |  | *0.9 (0.0, 1.8)* |  | *1.0 (0.2, 1.9)* |  | *0.8 (-0.1, 1.7)* |  | *0.6 (-0.4, 1.6)* |  | *1.3 (0.5, 2.1)* |  |
| **Five-year DSM** |  | 0.018 |  | 0.012 |  | 0.003 |  | 0.002 |  | 0.010 |  | 0.019 |  | <0.001 |
| 1 (Most affluent) | 1.00 (Ref.) |  | 1.00 (Ref.) |  | 1.00 (Ref.) |  | 1.00 (Ref.) |  | 1.00 (Ref.) |  | 1.00 (Ref.) |  | 1.00 (Ref.) |  |
| 2 | 0.96 (0.69, 1.32) |  | 1.05 (0.76, 1.44) |  | 1.08 (0.78, 1.48) |  | 1.12 (0.81, 1.54) |  | 1.08 (0.78, 1.49) |  | 0.98 (0.71, 1.35) |  | 1.06 (0.77, 1.45) |  |
| 3 | 1.28 (0.95, 1.74) |  | 1.38 (1.02, 1.87) |  | 1.40 (1.03, 1.90) |  | 1.48 (1.09, 2.01) |  | 1.46 (1.08, 1.98) |  | 1.44 (1.06, 1.95) |  | 1.43 (1.05, 1.94) |  |
| 4 | 1.11 (0.82, 1.50) |  | 1.21 (0.90, 1.63) |  | 1.25 (0.93, 1.68) |  | 1.33 (0.99, 1.78) |  | 1.24 (0.92, 1.66) |  | 1.25 (0.93, 1.69) |  | 1.31 (0.97, 1.77) |  |
| 5 (Most deprived) | 1.32 (1.00, 1.76) |  | 1.39 (1.05, 1.85) |  | 1.48 (1.11, 1.96) |  | 1.53 (1.15, 2.03) |  | 1.42 (1.07, 1.89) |  | 1.31 (0.99, 1.74) |  | 1.61 (1.20, 2.15) |  |
| *SII (95% CIs)* | *0.4 (-0.2, 1.0)* |  | *0.4 (-0.2, 1.0)* |  | *0.5 (0.0, 1.1)* |  | *0.6 (-0.1, 1.2)* |  | *0.5 (-0.3, 1.2)* |  | *0.4 (-0.4, 1.2)* |  | *0.7 (0.1, 1.3)* |  |
| **12-year DSM** |  | 0.005 |  | 0.005 |  | 0.001 |  | <0.001 |  | 0.003 |  | 0.005 |  | <0.001 |
| 1 (Most affluent) | 1.00 (Ref.) |  | 1.00 (Ref.) |  | 1.00 (Ref.) |  | 1.00 (Ref.) |  | 1.00 (Ref.) |  | 1.00 (Ref.) |  | 1.00 (Ref.) |  |
| 2 | 0.89 (0.66, 1.20) |  | 0.97 (0.72, 1.30) |  | 1.00 (0.74, 1.35) |  | 1.02 (0.76, 1.38) |  | 1.00 (0.74, 1.35) |  | 0.91 (0.67, 1.23) |  | 0.98 (0.72, 1.32) |  |
| 3 | 1.21 (0.91, 1.60) |  | 1.29 (0.97, 1.72) |  | 1.30 (0.98, 1.73) |  | 1.39 (1.04, 1.85) |  | 1.35 (1.02, 1.80) |  | 1.34 (1.01, 1.78) |  | 1.32 (1.00, 1.76) |  |
| 4 | 1.11 (0.84, 1.46) |  | 1.20 (0.91, 1.58) |  | 1.23 (0.94, 1.62) |  | 1.32 (1.00, 1.73) |  | 1.23 (0.93, 1.62) |  | 1.26 (0.96, 1.66) |  | 1.28 (0.97, 1.69) |  |
| 5 (Most deprived) | 1.30 (1.00, 1.70) |  | 1.35 (1.03, 1.76) |  | 1.43 (1.10, 1.86) |  | 1.48 (1.14, 1.92) |  | 1.39 (1.07, 1.81) |  | 1.30 (1.00, 1.69) |  | 1.51 (1.16, 1.98) |  |
| *SII (95% CIs)* | *0.4 (-0.1, 1.0)* |  | *0.3 (-0.2, 0.9)* |  | *0.6 (0.1, 1.0)* |  | *0.6 (0.1, 1.2)* |  | *0.5 (-0.1, 1.0)* |  | *0.4 (-0.3, 1.1)* |  | *0.7 (0.2, 1.1)* |  |

*Adjusted by age, sex and WHO Performance Status
^Adjusted by age, sex and smoking status
+Adjusted by age, sex and alcohol consumption
#Adjusted by age, sex and anatomical site
$Adjusted by age, sex and tumour stage
&Adjusted by age, sex and treatment modality
=Adjusted by age, sex and network

Supplementary Table 2 – All-cause mortality (ACM) and disease-specific mortality (DSM) hazard ratios adjusted by tumour or treatment factors for all patients

|  | **Adjusted by Tumour factors*** | | **Adjusted by Treatment^ factors^** | |
| --- | --- | --- | --- | --- |
| **Variable** | **HR (95% CIs)** | **p-value** | **HR (95% CIs)** | **p-value** |
| **One-year ACM** |  | 0.002 | 0.006 | 0.006 |
| 1 (Most affluent) | 1.00 (Ref.) |  | 1.00 (Ref.) |  |
| 2 | 1.32 (0.89, 2.95) |  | 1.18 (0.79, 1.74) |  |
| 3 | 1.53 (1.04, 2.25) |  | 1.55 (1.06, 2.29) |  |
| 4 | 1.53 (1.06, 2.21) |  | 1.51 (1.04, 2.18) |  |
| 5 (Most deprived) | 1.73 (1.22, 2.46) |  | 1.59 (1.11, 2.29) |  |
| *SII (95% CIs)* | *0.8 (0.2, 1.3)* |  | *0.7 (0.0, 1.4)* |  |
| **Five-year ACM** |  | 0.004 |  | 0.003 |
| 1 (Most affluent) | 1.00 (Ref.) |  | 1.00 (Ref.) |  |
| 2 | 1.03 (0.81, 1.31) |  | 0.90 (0.71, 1.15) |  |
| 3 | 1.32 (1.04, 1.66) |  | 1.24 (0.98, 1.57) |  |
| 4 | 1.13 (0.90, 1.42) |  | 1.14 (0.91, 1.43) |  |
| 5 (Most deprived) | 1.33 (1.08, 1.65) |  | 1.28 (1.03, 1.59) |  |
| *SII (95% CIs)* | *0.4 (-0.2, 0.9)* |  | *0.4 (-0.1, 1.0)* |  |
| **12-year ACM** |  | 0.015 |  | 0.015 |
| 1 (Most affluent) | 1.00 (Ref.) |  | 1.00 (Ref.) |  |
| 2 | 0.95 (0.78, 1.16) |  | 0.87 (0.71, 1.06) |  |
| 3 | 1.15 (0.94, 1.40) |  | 1.10 (0.91, 1.34) |  |
| 4 | 1.07 (0.88, 1.29) |  | 1.07 (0.89, 1.30) |  |
| 5 (Most deprived) | 1.19 (0.99, 1.42) |  | 1.14 (0.95, 1.37) |  |
| *SII (95% CIs)* | *0.3 (-0.1, 0.6)* |  | *0.3 (-0.1, 0.6)* |  |
| **One-year DSM** |  | 0.013 |  | 0.012 |
| 1 (Most affluent) | 1.00 (Ref.) |  | 1.00 (Ref.) |  |
| 2 | 1.54 (0.96, 2.48) |  | 1.36 (0.85, 2.18) |  |
| 3 | 1.69 (1.06, 2.71) |  | 1.77 (1.11, 2.84) |  |
| 4 | 1.64 (1.04, 2.58) |  | 1.67 (1.06, 2.63) |  |
| 5 (Most deprived) | 1.85 (1.20, 2.86) |  | 1.79 (1.15, 2.80) |  |
| *SII (95% CIs)* | *0.8 (-0.1, 1.7)* |  | *1.1 (-0.2, 2.4)* |  |
| **Five-year DSM** |  | 0.014 |  | 0.001 |
| 1 (Most affluent) | 1.00 (Ref.) |  | 1.00 (Ref.) |  |
| 2 | 1.10 (0.80, 1.51) |  | 0.96 (0.69, 1.32) |  |
| 3 | 1.52 (1.12, 2.06) |  | 1.47 (1.09, 2.02) |  |
| 4 | 1.26 (0.93, 1.70) |  | 1.34 (0.99, 1.80) |  |
| 5 (Most deprived) | 1.42 (1.07, 1.88) |  | 1.46 (1.09, 1.95) |  |
| *SII (95% CIs)* | *0.4 (-0.4, 1.3)* |  | *0.6 (-0.2, 1.4)* |  |
| **12-year DSM** |  | 0.005 |  | <0.001 |
| 1 (Most affluent) | 1.00 (Ref.) |  | 1.00 (Ref.) |  |
| 2 | 1.01 (0.75, 1.36) |  | 0.89 (0.66, 1.21) |  |
| 3 | 1.40 (1.05, 1.87) |  | 1.37 (1.03, 1.83) |  |
| 4 | 1.25 (0.94, 1.64) |  | 1.32 (1.00, 1.74) |  |
| 5 (Most deprived) | 1.38 (1.06, 1.79) |  | 1.38 (1.05, 1.82) |  |
| *SII (95% CIs)* | *0.5 (-0.2, 1.1)* |  | *0.6 (-0.2, 1.3)* |  |

*Adjusted by age, sex, anatomical site and tumour stage
^Adjusted by age, sex, treatment modality and network
